# The mismatch in hierarchical diagnosis and treatment construction and related reasons: a qualitative analysis from ZX City, Hubei Province

**DOI:** 10.3389/fpubh.2025.1524732

**Published:** 2025-08-21

**Authors:** Yuhui Ruan, Mingming Yu

**Affiliations:** ^1^School of Politics and Public Administration, Soochow University, Suzhou, Jiangsu Province, China; ^2^Department of Economics and Management, Shanghai Technical Institute of Electronics & Information, Shanghai, China

**Keywords:** mismatch, hierarchical diagnosis and treatment construction, difficult and expensive access to medical treatment, initial diagnosis at the grassroots level, allocation of health resources

## Abstract

**Background:**

There is a substantial gap between the objective of hierarchical diagnosis and treatment (HDT) construction and its actual effectiveness in resolving the problems of difficult and expensive access to medical treatment. Consequently, it has become essential to address these issues through research.

**Objectives:**

This study intends to examine the mismatch in China’s HDT construction and identify its underlying causes.

**Methods:**

Grounded theory was employed in this research. Open questions were designed through theoretical sampling and coding processes until theoretical saturation was achieved. A total of 52 participants, all doctors from different levels of medical institutions in ZX City, Hubei Province, China, with more than 3 years of experience, were recruited.

**Results:**

This study uncovers significant mismatches between residents’ “pursuit of high-quality medical care” and HDT’s requirement of “initial diagnosis at the grassroots level,” between the service supply capacity and the functional positioning of medical institutions in HDT, and between the policy environment and HDT’s construction needs, respectively. The primary reasons for these discrepancies are residents’ lack of confidence, improper allocation of health resources, and the absence of in-depth health policy reforms.

**Conclusion:**

This study recommends restoring residents’ confidence in the service provision of low-level medical institutions to achieve initial diagnosis at the grassroots level, establishing a positive health resource–allocation pyramid from lower to higher levels to enhance the service capacity of primary health care institutions, and adjusting the policy environment to address and reform deep-seated policy contradictions and rationalize the functional positioning and matching relationship of medical institutions at all levels in HDT.

## 1 Introduction

Since the initiation of the healthcare system reform in 2009, China has implemented and promoted “Hierarchical Diagnosis and Treatment” (abbreviated as “HDT” hereinafter) to effectively integrate health resources, rationally optimize diverse health demands, and efficiently tackle the challenges faced by healthcare reform (1). In the years since, a series of new healthcare reform policies centered on the policy basis of HDT construction have been introduced. In 2015, the General Office of China issued the Guiding Opinions on Promoting the Construction of Graded Diagnosis and Treatment System, which put forward the connotation of HDT in China based on clarifying the functional position of diagnosis and treatment services of various types of medical institutions at all levels (2)—namely, “grassroots first diagnosis and treatment, two-way referrals, separation of emergencies and chronic diseases, and up-and-down linkage” (3). The document advocates for focusing on “strengthening the grassroots” and promoting the formation of an orderly medical treatment pattern where “minor illnesses are treated in the community, major illnesses are treated in hospitals, and rehabilitation is carried out at the grassroots.” Nevertheless, the effectiveness of HDT in resolving the problems of “difficult and expensive access to medical treatment” remains unsatisfactory (3). High-level medical institutions are still overcrowded, while low-level ones are largely underutilized (4). The supply and demand of health services in China exhibit a significant mismatch with the goal of HDT. However, there has been insufficient research on the manifestations and causes of this mismatch, along with the potential solutions to address the exposed dilemmas. Consequently, resolving these issues through research has become a key focus for scholars.

In China, the HDT system primarily classifies medical institutions based on the severity, urgency, and difficulty of diseases for treatment and diagnosis (5, 6). Different levels of medical institutions are assigned, with corresponding treatment responsibilities. By setting clear goals and well-defined powers and responsibilities for each institution, a fair and effective division of labor and cooperation is ensured, ultimately facilitating the gradual transition of patients from general practitioners (GPs) to specialized treatments (7). Based on their technical level and size, medical institutions in China are categorized into three levels (8). The primary level includes grassroots hospitals that directly provide comprehensive medical services, including preventive care, rehabilitation programs, maternal–child healthcare services, and family planning initiatives within communities. According to the systematic arrangement of HDT, initial diagnoses for minor illnesses (mainly common and chronic conditions) should be conducted at primary-level (first-level/primary/grassroots) medical institutions, such as community hospitals/health service centers/stations, which is known as “primary care at the grassroots level” (9). Services for general diseases, like general surgical treatment and more common and difficult diseases, are the responsibility of second-level medical institutions. Tertiary medical institutions mainly offer specialized medical services for critical and difficult illnesses, major diseases, and rare diseases, and they are also subject to secondary referrals (5, 6). Additionally, they provide operational and technical guidance as well as training opportunities to lower-level medical institutions (10). Clearly, the HDT system differentiates medical institutions at different levels with distinct functional positions. Within this framework, each institution is required to fulfill its designated functions while collaborating smoothly to meet the diverse healthcare needs of the public.

To effectively triage the supply of medical services and circumvent excessive competition, the majority of developed nations have established relatively standardized systems—namely, integrated healthcare systems (11), medical referral systems (11), and “gatekeeper” mechanisms (12). Commonly, HDT places emphasis on prioritizing the utilization of primary health services. Through the guidance of GPs, patients truly requiring referrals or specialized treatments can be more appropriately matched with relevant resources in specialty fields (13). This approach contributes to reduced healthcare costs and enhanced efficiency of specialty resource utilization. To attain these goals, some countries capitalize on the affordability of primary healthcare services to draw patients toward primary health services voluntarily. However, the majority of others adopt mandatory measures, mandating all (or publicly insured) residents to obtain a GP referral before accessing specialized care (14). Primary medicine mainly constitutes outpatient services offered by GPs, while secondary medicine encompasses consultations with specialized doctors for complex and variable conditions, critical cases, major ailments, or those in need of specialized treatment (14). Obviously, promoting effective collaboration between primary healthcare institutions and higher-level ones by HDT can positively impact the effectiveness of health services (15).

In many developing countries, mature and standardized HDT systems are still lacking. The allocation of resources is mainly concentrated in specialty and hospital systems, resulting in fragile primary healthcare resources and low utilization rates (2, 16). China has initiated the construction of the HDT system for a relatively short duration; as such, the majority of efforts remain exploratory and in need of further refinement (8, 17). Currently, numerous disparities exist in the construction of HDT in China. Despite the considerable progress achieved by China in the infrastructure development of primary medical institutions since 2003, especially after the new round of healthcare reforms, the reform of the service delivery system still lags behind (18). Although considerable attention has been paid to accessibility for determining the referral rate between hospitals, there is a widespread lack of cooperation and integration between low-level and higher-level institutions, giving rise to fragmented services that have a negative impact on performance and cost control (19). Among these challenges, the delayed implementation of payment reforms and the shortage of high-quality primary care physicians, particularly in rural areas, are currently regarded as extremely serious (20, 21). Additionally, the regulatory role of health departments and the enhancement of the participation of medical institutions and patients are considered of vital importance (15).

The construction of HDT is considered to be a systematic project since it involves multiple subjects and interests (10, 22, 23). Whether it is the requirement of a first consultation for residents or the functional positioning of medical institutions at all levels within the HDT system, its various aspects are aimed at achieving a better match between supply and demand (10, 24). The mutual matching of elements serves as the key to attaining coordination and coherence among different medical supply and demand (24). Coordination and coherence among the subjects of HDT are of crucial significance for the attainment of policy goals (25, 26). To objectively analyze the matching relationship within the medical service system, the matching of supply and demand has consistently been a focus area (27).

Current research has already conducted extensive and in-depth investigations into HDT construction. It has explored aspects such as the status of necessary resource allocation, its operation, and the consequences and causes of distortion (22, 28, 29). Existing studies have also focused on integrating existing medical resources to enhance their utilization efficiency, especially strengthening the service functions of primary medical institutions, achieving vertical integration among multi-level medical institutions, and establishing a gatekeeper system (30, 31). Generally, there is considerable research on resource integration for efficiency improvement. However, insufficient studies have explored the mismatch in China’s HDT construction and its causes, resulting in a lack of a sound judgment basis for improvement strategies. Clearly, clarifying this mismatch and its causes is crucial for resolving issues like disordered medical care, resource insufficiency and waste, and high medical costs in China. Hence, a qualitative study has been formulated to address these matters and is reported herein.

## 2 Method

### 2.1 Study design

**Figure 1:**
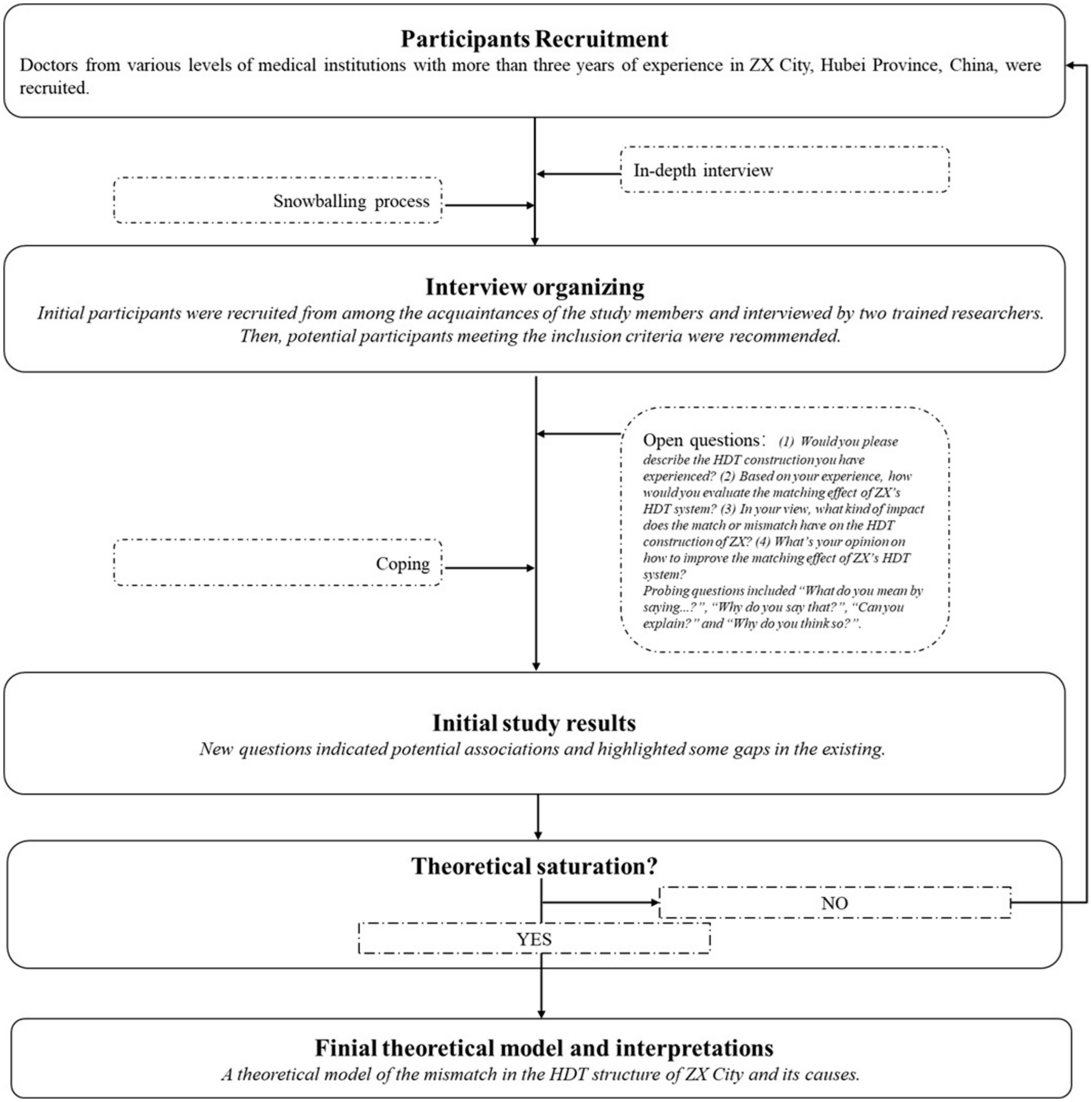
Study design. This figure depicts the iterative research process implemented in ZX City, Hubei Province. First, purposive sampling of licensed physicians with at least 3 years of post-qualification experience was performed across tertiary, secondary, and primary public facilities. Second, snowball recruitment through the professional networks of the initial informants was carried out to reach under-represented strata. Third, interviews using an interview guide with open-ended and probing questions focused on perceived effectiveness, barriers, and facilitators of the HDT system were conducted. Fourth, continuous concurrent analysis was completed. Theoretical saturation was achieved at a final sample of *n* = 52.

To examine the mismatch in China’s HDT construction and uncover its causes, a qualitative study by mixing in-depth interviews and grounded theory approaches has been formulated. Participants were recruited via a snowballing process, and the data were mostly recorded from in-depth interviews. Open-ended questions for in-depth interviews were designed and refined through a pre-survey. All questions and analyses were ongoing, focusing on the mismatch of HDT and its reasons. Finally, all data were continuously collected and analyzed in accordance with the grounded theory method, as depicted in Figure 1.

Grounded theory is a systematic methodology for developing theories that emphasizes inductive analysis. It is known to provide an advantage relative to normative approaches in developing new theories or hypotheses. Thus, grounded theory was deemed appropriate because of its data-driven orientation (32). A grounded theory approach was adopted in this study to enrich the data of the in-depth interviews and help explore how to recognize and guide the mismatch of HDT and its reasons from the perspective of various providers. In-depth interviews can be used to better understand some special questions (33), such as those concerning the experiences, perceptions, and views of samples (34), hence the combination of the two methods.

### 2.2 Participants

#### 2.2.1 Inclusion criteria

Participants were recruited from ZX, Hubei Province, China. ZX has two municipal hospitals, two specialized hospitals, and six township hospitals. On January 31, 2015, ZX initiated a pilot HDT system; as of September 1, 2016, its HDT system has been fully implemented. However, to date, the construction effect remains unsatisfactory. The development of HDT in ZX is generally representative, covering aspects such as medical resource allocation, referral mechanisms, inter-agency collaboration, patient policy implementation, and policy guidance. Hence, this study chose to conduct a survey in this region to ensure that the research results would be more widely representative. Doctors from all levels of medical institutions with more than 3 years of experience were included. Other inclusion criteria included that participants should not have any communication difficulties and must be willing to share their opinions. A total of 52 participants were recruited for this study.

#### 2.2.2 Transferability statement

To facilitate external comparisons, the contextual parameters of ZX City are summarized below. According to the 2023 Health Statistical Yearbook of Jingmen Municipality, ZX City had a resident population of 867,000 and a healthcare infrastructure density of 5.1 beds and 2.1 practicing (assistant) physicians per 1,000 inhabitants. The HDT pilot was launched in 2016, and the county-level integrated medical community has been fully operational since 2019. Household doctor contracts cover 100% of key populations—defined as older adults, individuals with chronic diseases, and low-income residents. Researchers wishing to transfer these findings should therefore assess the alignment of their settings with these benchmarks. Substantial deviations in population size or policy maturity may constrain direct generalizability.

#### 2.2.3 Sampling process

The sampling process was carried out from July 1, 2023, to September 30, 2023. Initially, participants were recruited from among the acquaintances of the study members and interviewed by two trained researchers. Subsequently, potential participants who met the inclusion criteria were referred for participation in this study. All participants were informed in detail about the purpose and process of the study. They were included only after confirming their full understanding and willingness to participate in the study (through oral or written informed consent). In-depth interviews with each participant were conducted in separate spaces to ensure privacy and concentration. Each interview lasted approximately 60–90 min and was transcribed and recorded by trained researchers. At the end of each interview, participants were encouraged to recommend other suitable individuals to join the study. Post-study debriefing reports can be offered to all participants depending on their request. Anonymous references (letters and numbers) were used to ensure the confidentiality of the participants (as shown in Table 1). All procedures strictly adhered to institutional ethical standards for non-clinical industrial studies at Soochow University.

**Table 1:** Basic information statistics of the participants in this study.

	Number	Percentage (%)
Age (years)		
<40	18	34.6
40–50	21	40.4
>50	13	25.0
Gender		
Female	24	46.2
Male	28	53.8
Education		
Below bachelor’s degree	5	9.6
Bachelor’s degree	39	75.0
Master’s degree and above	8	15.4

### 2.3 Data collection

This research endeavor was instigated by an as-yet-unaddressed query: “How is the mismatch in ZX’s HDT construction and what are the corresponding factors?” In the discovery phase, the feedback obtained from in-depth interviews, responses from participants, and the interviewer’s perceptions and ruminations were meticulously documented and analyzed. Subsequently, through a stringent process of analysis, coding, and memo generation, specific conceptual categories were formulated for the hitherto unexplored issues—primarily “mismatch in ZX’s HDT construction” and “corresponding factors.” This inductive analytical procedure enabled the emergence of key issues through continuous interviews and induction. Grounded in the research methodology of grounded theory, the final conclusion was derived from the specific problem to the more general realm.

The open-ended questions designed for the in-depth interview are as follows: (1) Would you please describe the HDT construction you have experienced? (2) Based on your experience, how would you evaluate the matching effect of ZX’s HDT system? (3) In your view, what kind of impact does the match or mismatch have on the HDT construction of ZX? (4) What’s your opinion on how to improve the matching effect of ZX’s HDT system? All these open questions were seamlessly integrated into the conversation. As the interviews progressed, trained researchers responded by asking more probing questions, such as “What do you mean by saying?,” “Why do you say that?,” “Can you explain?,” and “Why do you think so?” Participants were also encouraged to provide more details about their views by using expressions like “Could you give me more details about?”

A multi-step verification protocol was employed. First, two researchers independently open-coded the first round of interview transcripts, and the inter-coder reliability reached 0.83 (Cohen’s kappa). During the analysis, discrepancies were resolved through negotiated agreements, and the codebook was updated iteratively. Second, methodological triangulation was achieved by integrating participant narratives, policy documents, and detailed field notes, which corroborated or contextualized the emergent categories. Finally, member checking was conducted with three key informants, who confirmed 87% of the conceptual categories, thereby supporting the credibility of the findings. The data-collection process proceeded through in-depth interviews until there was scarcely any new information to be obtained. To be detailed, in the final data-collection process, saturation was determined through systematic assessment; notably, data saturation was confirmed when three consecutive interview batches each generated no more than 5% additional codes beyond the existing coding frame, prompting the termination of new in-depth interviews. Regular group discussions were organized among researchers to facilitate the timely exchange of information and the sharing of emerging ideas.

### 2.4 Data analyses

Data analysis was initiated following the completion of the initial two interviews. To maximize the extracted information, an open coding approach was employed. Verbatim survey transcripts, memos, and other documents were read and analyzed. A combination of line-by-line and sentence-by-sentence encoding was frequently implemented. Additionally, integrative coding of sentences or even paragraphs with similar or related meanings was permitted when appropriate. Through this process, higher-level concepts were abstracted and labeled (as codes). Simultaneously, a multi-level coding procedure was carried out wherein the aforementioned codes were continuously modified, integrated, and even discarded as subsequent materials were added. Through stepwise analysis of the data, the research content was gradually clarified, and the concepts and categories were elucidated. Labels were repeatedly considered and compared throughout the coding process.

As explained, this study was carried out by simultaneously employing open and focused coding. Initial categories were generated through the initial grouping of the first batch of codes. These initial open categories exerted an influence on the subsequent sampling process and guided the ensuing interviews and observations. Once the initial sampling preference was formed, researchers paid increased attention to when a mismatch existed (including mismatches between “chasing high-level medical [services]” and “[being] initially diagnosed [at the] grassroots [level]” and between “service supply capacity” and “functional positioning” in HDT, along with reasons for the mismatch) and to “the policy environment and HDT requirements” (including “[a] lack of confidence,” “unreasonable allocation of health resources,” and “deep-rooted health policy reforms not yet in place”). As the sampling process advanced, the categories, attributes, and dimensions of some of the outlines and guidelines became more explicit. Later on in the analysis, a theoretical model of the mismatch(es) in the HDT structure of ZX and its cause(s) was initially put forward. The composition, nature, and characteristics of the theoretical model lie in the fact that certain properties and dimensions, as well as names and categories, were constantly reinforced during the course of the study.

## 3 Results

**Figure 2:**
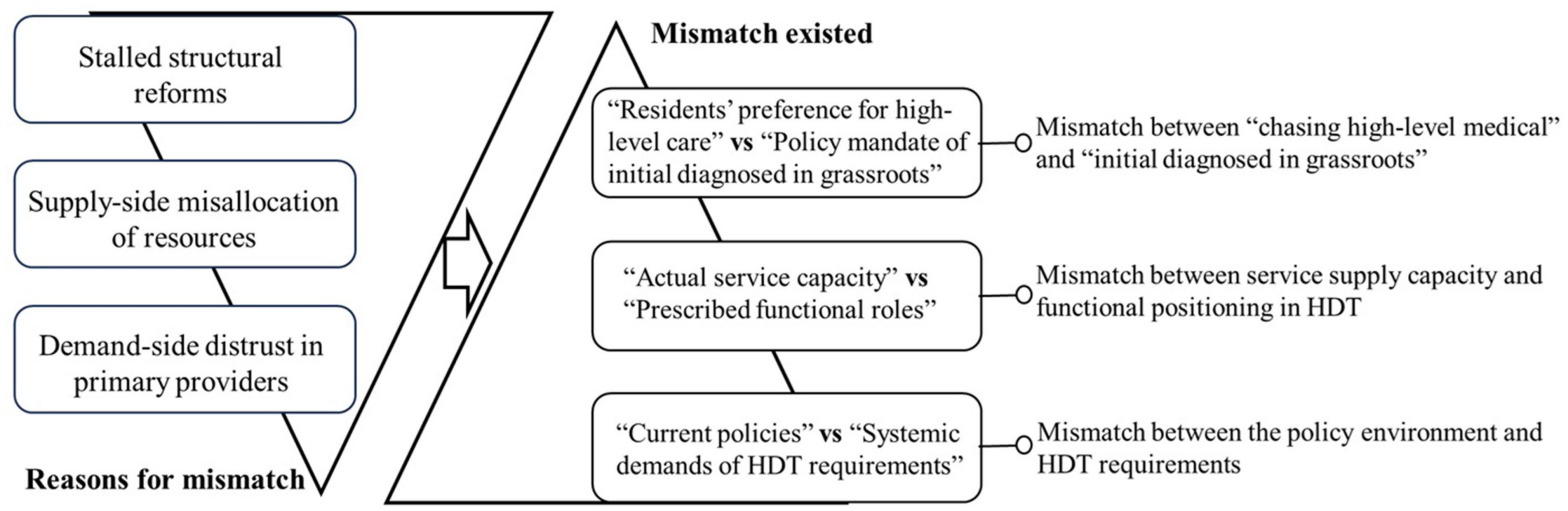
Conceptual diagram. As shown, reasons like demand-side distrust, supply-side misallocation, and stalled reforms have jointly resulted in current systemic mismatches between patient preference and policy, service capacity and role prescription, and policy environment and HDT requirements, respectively.

This study uncovered a pervasive, three-dimensional mismatch within ZX’s HDT system, as shown in Figure 2. Specifically, the mismatches manifest in three aspects: residents’ preference for high-level care mismatching with the policy mandate of being initially diagnosed at the grassroots level, actual service capacity mismatching with the prescribed functional roles, and current policy environment mismatching with the systemic demands of HDT requirements. Further analysis indicated that these mismatches are driven by the demand side’s distrust in primary providers, the supply side’s misallocation of resources, and stalled structural reforms. These reasons have driven the existing misalignments jointly and currently undermine overall HDT’s efficiency.

### 3.1 Mismatches exist

Mismatch is common in HDT. Residents’ pursuit of high-level medical services is a widespread phenomenon. The service supply capacity of medical institutions at all levels in the HDT system typically fails to match their functional positioning. Also, the existing policy environment is still unable to meet the demands of HDT construction.

#### 3.1.1 Mismatch between “chasing high-level medical” and “initially diagnosed at the grassroots”

Driven by residents’ diverse service demands, residents’ behavior of “chasing high-level medical [services]” is mismatched with the requirements of HDT, particularly with the principle of “[being] initially diagnosed [at the] grassroots [level].”

How can policy requirements like “[being] initially diagnosed [at the] grassroots [level]” be transformed into behavioral constraints for residents? This is a challenging problem. Currently, residents’ behavior is mainly driven by their demands for health services. There are still relatively few effective policy constraints on their behavior. This gives rise to the phenomenon of chasing high-level medical care. (JK-2)

“Chasing high-level medical [services]” is utilized by the participants to describe the scenario wherein patients with common and chronic diseases do not seek initial diagnosis at the grassroots level but instead directly proceed to high-level medical institutions. This is perceived as the result of a “mismatch between medical supply and demand.” It is also deemed closely related to the fact that “China’s HDT system is not based on a strict gatekeeper system at the grassroots level.” As a consequence, the flexibility in accessing medical services at all levels is not constrained by the HDT policy. Although some supporting measures, such as the “15-min health service circle,” lower registration fees, and higher reimbursement rates, are intended to guide residents’ “initial diagnosis at the grassroots level” through “proximity” and “low costs,” they appear to be ineffective. On the contrary, due to their “excessive concern for health,” residents tend to adopt the strategy of “chasing high-level medical [services]” in this process, rather than pursuing “initial diagnosis [at the] grassroots [level]” as expected by HDT.

Instead of guiding residents to get an initial diagnosis at the grassroots level, the hierarchy of medical institutions has become a standard for many residents to judge the quality of the services provided. The services provided by high-level medical institutions are generally thought to be better. So, even if residents know the policy requirements of HDT, many of them still won’t choose to have an initial diagnosis at the grassroots level and insist [instead] on going directly to a high-level medical institution. (CH-5-)

I believe that for the vast majority of people, everything else can be a bit slack, but not their health. If you can go to a tertiary hospital to get health services (meaning, as long as the policy doesn't stop you), then you’ll still go to the tertiary hospital. Patients are not rational and seem to care less about the cost or the distance in this process. (YZ-1)

Residents’ “chasing high-level medical [services]” behavior significantly contributes to the mismatch between health service demand and supply. In practice, many common services of low-level medical institutions are also provided by high-level ones. Driven by profit temptations and the mission to “save lives and help the sick,” high-level institutions will not and cannot refuse patient demands, even for services supposed to be offered at the low level. Meanwhile, despite not aligning with the “[being] initially diagnosed [at the] grassroots [level]” policy of HDT but still meeting residents’ needs, this behavior causes a supply–demand mismatch. As a result, many residents do not go to grassroots institutions, leading to overcrowding in high-level ones, a waste of high-quality resources on common diseases, under-utilization of low-level services, and a waste of primary medical resources. Moreover, “chasing high-level medical [services]” weakens the incentive for inter-level medical institution collaboration, contrasting with the HDT policy goals.

Those high-level medical institutions are still really crowded. Even so, they won’t refuse to provide basic medical services just because they’re supposed to be the responsibility of the lower-level ones. After all, medical institutions also have the aim of making money. What’s more, refusing to provide services might lead to doctor–patient conflicts. They simply wouldn’t dare. (LS-3)

For the residents, even if their behaviors don’t comply with the policy requirements, they can still be satisfied accordingly in high-level medical institutions. As long as the HDT policy requirements aren’t mandatory, their behavior of going after high-level medical care is free and unrestrained. (YZ-2)

#### 3.1.2 Mismatch between service supply capacity and functional positioning in HDT

The service supply capacity of medical institutions at all levels fails to align with their functional positioning within the HDT framework. Particularly, the service supply capacity of low-level medical institutions is insufficiently high to match their functional positioning.

Generally speaking, the supply of primary health services is still not enough. Whether in terms of quality, quantity, or variety, there's a clear gap between them and the current demand for residents' health services. Generally, their service supply capacity doesn't match up with their functional positioning in the HDT system. (YZ-6)

The service provision of low-level medical institutions, especially at the grassroots level, is misaligned with the residents’ demands. For instance, a mismatch is caused by service coverage, like the narrow range of covered disease types, medicines, and the limited number of residents. There is also a mismatch attributed to service capacity, which includes restricted medical technology, an inferior environment, and inadequate hardware and software facilities. Furthermore, a mismatch is brought about by service effect, such as insufficient preventive education, unsatisfactory health care results, and poor health management. Currently, primary health services have long shifted toward emphasizing the prevention and treatment of geriatric and chronic diseases, especially consumptive health services like common and frequent diseases. Nevertheless, the grassroots lag behind in making adaptive adjustments to their health services supply. There still exist deficiencies in the prevention, treatment, and management of relevant diseases. As a result, the supply capacity of the grassroots still fails to correspond with their functional positioning in the HDT.

The role of the grassroots in HDT is to provide wide health services supply coverage for common/frequent diseases that have a high incidence rate and a long treatment cycle but don’t need advanced technical skills. However, this requirement is still a bit too much for many of our current primary medical institutions. (SP-4)

The supply of services at our grassroots hasn’t improved a lot for many years. But you need to understand that the types of diseases and the needs of the population have actually been changing. Therefore, it’s still very difficult for the current supply of primary health services to adapt and match its functional position in HDT. (RM-1)

The current supply of primary health services has not achieved “quality coverage of rural and grassroots communities.” In many rural and community areas, the shortage of doctors and medicine has not been fully reversed yet. Regarding the gap in grassroots institutions’ health service supply, the corresponding demand undoubtedly flows into high-level medical institutions. This intensifies both the pressure on high-level medical institutions’ service provision and residents’ medical costs, shown as “difficult access to healthcare” and “expensive access to healthcare.” Clearly, this is the problem HDT is striving to solve. However, due to the mismatch between grassroots’ supply capacity and functional positioning, the above problems have not been effectively addressed. Meanwhile, although high-level medical institutions’ functional positioning in HDT is to “mainly treat critical and complex diseases,” they still admit and treat a large number of common and frequent disease patients in practice. This is a departure from their positioning and another difficulty in matching HDT’s goals.

If the service provision at the grassroots [level] doesn't match up with its functional positioning in HDT, residents will naturally head for high-level medical institutions. And then, “difficult access to health care” and “expensive access to health care” still haven’t been sorted out. Obviously, this mismatch has seriously affected the construction result of HDT. (DQ-4)

High-level medical institutions also provide services that are within the functional positioning of low-level’s in HDT. Currently, a large amount of high-quality health resources in high-level medical institutions have been put into basic health services. Not only are high-quality resources being wasted, but medical resources at the primary level are also being left idle and wasted. (CH-1)

#### 3.1.3 Mismatch between the policy environment and HDT requirements

The current health policy environment is incongruous with the policy requisites of HDT. The aim of HDT is to optimize the allocation of health resources and accomplish the division of labor and collaboration among medical institutions at all levels. Nonetheless, a significant number of the existing policy guidelines do not match these requirements.

Healthcare reform has an impact on the whole health system. The issues of “difficult access to health care” and “expensive access to health care” aren’t the result of any single policy/measure, and they won’t be fixed just by the policy/measure of HDT either. A lot of other mismatched policies need to be adjusted. (YZ-3)

HDT is widely acknowledged as “one of the most crucial reform measures of health care reform.” It’s also seen as “a reform based on the existing policy environment.” Regrettably, most participants believe that there is misalignment between it and the current policy environment in that it has been significantly constrained. Although low-level medical institutions (particularly grassroots ones) have developed to some extent under HDT’s policy guidance, they still lag behind high-level ones, which is regarded as a consequence of the policy environment. For instance, due to administrative level disparity, high-level medical institutions have gained far more support. Simultaneously, institutional arrangements for personnel and benefits are unreasonable, causing misalignment. For example, individuals with top-quality talents and skills find it difficult to flow, as they are constrained by the existing establishment system and the welfare system attached to it, as well as the scope of practice. It is challenging for these health practitioners to move downward. Another example is that the reform of the basic drug system and the financial and expenditure systems has dampened the motivation of primary medical institutions and health service personnel. All the aforementioned factors have resulted in the strong enrichment capacity of high-level medical institutions in terms of health resources and have become a key reason why primary medical institutions have been unable to grow stronger and larger for a prolonged period.

Even though HDT is well planned, … in the current policy environment, as long as the gap in the allocation of health resources hasn't been turned around, the grassroots won't become stronger. Since the policy of allocating resources based on administrative levels stays the same, high-level medical institutions will just keep getting more powerful. (JK-6)

The pensions, title evaluations and welfare benefits related to the establishment of high-level medical institutions have seriously stopped the outflow of high-level talents and strongly attracted outstanding ones to join. The two-line system of income and expenditure, together with the basic drug system, have further lowered the work motivation of primary health service personnel. (ZY-3)

Many participants believed that “the goal of HDT is to establish a pyramid-shaped health service system with a narrow top and a wide base.” The function of primary medical institutions at the base of such a “pyramid” is to “provide the largest number of services to the largest number of residents.” This is the means to achieve “maximum coverage” and the effective provision of health services. The emphasis of high-level medical institutions should be on technical upgrading rather than quantitative expansion. Then, the HDT system can effectively cover various service provisions by establishing a division of labor and facilitating smooth upward and downward referral pathways. However, in the current policy environment, in many regions, there is continuous investment in the construction of high-level medical institutions in a “blind competition.” Numerous high-level medical institutions are also constantly expanding the scale of their resource inputs, including the recruitment of talent, the number of beds, and the construction of hospital districts. In contrast, low-level medical institutions, which have a greater need to expand their capacity, are overburdened and lack policies that align with their needs. Participants even noted, “many misalignments result from the policies carried over from the first round of healthcare reform.” If they cannot be adjusted, it will be difficult to realize the policy objective of HDT.

We’re not rebuilding. We’re reforming and adjusting based on the existing policy environment. You’ve got to realize that a lot of the current unreasonable situations are the results of the policies that are already in place. Especially, many policies from the first round of health care reform have had a deep impact on the current HDT. For example, the allocation of health resources, right? There’s a clear mismatch. (JK-1)

I”s really hard for the low-level medical institutions to fulfill their functions. After the first round of health care reform, the health care staff there dwindled and the equipment got old. Even though about another decade has passed since the second round of health care reform, as long as there’s not much important change in the policies for talents, there still won’t be anyone coming. (ZY-3)

### 3.2 Reasons for the mismatch

The mismatch above largely results from issues on both the supply and demand fronts. On the demand side, residents lack confidence in primary medical institutions. On the supply side, there is improper resource allocation within medical institutions. Additionally, deep-seated problems remain unresolved in the policy environment of healthcare system reform.

#### 3.2.1 Lack of confidence

Residents face difficulty in accumulating adequate confidence in the health service provision of low-level medical institutions. They are not regarded as capable of delivering quality services that truly cover common and chronic diseases. Participants view residents’ lack of confidence not as a simple unfriendly bias but as an objective conclusion based on long-term life experiences, including personal consultation and factual ones. Since the construction of HDT, while some changes have occurred in health service provision at low-level institutions, the essential aspects have not changed much. Negative experiences and news have constrained residents’ confidence in the current low-level health services. As a result, many residents remain reluctant to implement the “initial diagnosis in grassroots” rule as required by HDT and insist on “chasing high-level medical care.”

For years, residents have gotten a bad impression of the health service provided by low-level medical institutions. It’s really hard to convince them to have enough confidence in a short time. (ZY-1)

The service provision capacity of low-level medical institutions is indeed limited. Their supply capacity in terms of treating the sick and saving lives is still not convincing. If ask the residents to perform “initial diagnosis in grassroots”, you are asking them to use services in which they have no confidence. (YZ-7)

Objectively, there is a substantial disparity in the provision of health services across all levels of medical institutions in China. It is essentially an indisputable fact that “high-level medical institutions equate to high-quality service provision.” Consequently, residents are more inclined to trust the services provided by high-level institutions. In reality, HDT is more reliant on a robust primary health care system, which forms the basis for a synergistic “primary care and two-way referral” scenario. However, participants have still pointed out that “the actual referral channel is not entirely smooth.” The main challenges include “the procedures being still complex” and “low patient willingness.” The “revealed referral issues” have also created numerous negative experiences for residents and diminished their confidence. Meanwhile, competition for service users between low-level and high-level institutions is inevitable. Confidence in service provision is an important means of competition. Therefore, high-level medical institutions are continuously optimizing the quality of their service provision to consolidate more residents’ confidence. Due to their technological and resource superiority, they are more capable of winning residents’ confidence than grassroots institutions. As a result, the confidence gap between different levels of medical institutions among residents is effectively maintained over time. This is regarded as one of the main reasons for the mismatch between “chasing high-level medical care” and “initial diagnosis in grassroots.”

A division of labor among different levels of medical institutions is required. However, medical institutions are more in need of being self-sustaining. As a result, it’s tough for them to cooperate synergistically. To some extent, they are still in a certain degree of competition. In this competition, residents tend to have more trust in high-level medical institutions. (YZ-4).

Currently, residents are really distrustful of grassroots institutions. They know very well how bad the services there are. In their opinion, for services that grassroots institutions could not provide before the construction of HDT, they still cannot provide now. So, as long as residents do not trust them, the mismatch between “going after high-level medical care” and “getting initially diagnosed at grassroots level” will not be resolved. (JK-5).

#### 3.2.2 Unreasonable allocation of health resources

“Extensive coverage of common and frequent diseases” highly demands the provision of health services by low-level medical institutions. They are required to offer residents “longer-term follow-up and observation”; “regular communication”; and “physical and psychological counseling, including interpersonal counseling.” This involves “establishing a good relationship of trust with patients,” “establishing information communication and interaction based on trust,” and “establishing a standardized patient resume and health management process.” To match these functions, grassroots institutions require a large number of physicians with multidisciplinary knowledge backgrounds. For a long time, high-level medical institutions have been flush with a large pool of highly skilled personnel, have had access to advanced health service technologies, and have been at the forefront of healthcare information and communication. In contrast, lower-level medical institutions have developed slowly due to the lack of sufficient technical and financial support and, as a result, residents find the quality of their health services provision more questionable. Although HDT has continuously improved the supply capacity of grassroots institutions, the content and form of services at the grassroots level are still under construction, and the results are not obvious. Meanwhile, the development of new service contents requires efforts based on multiple resources, and it is still difficult for grassroots-level institutions to accomplish. Therefore, as of now, the health services provision of grassroots institutions still fails to match their functional positioning.

Our communities have completed the “project of contracted family doctors.” But, there’s still a long way to go. This project has just started and hasn’t been able to provide more practical services like health management and health planning for residents yet. Once residents need health services, they are likely to be on their own still. (SP-3)

To match their functional needs in HDT, one of the main difficulties for lower-level medical institutions is the lack of GPs. But it takes ten years or more to train a GP. This is not counting the time gap between policy planning and actual implementation. Therefore, for a long time, the health service capacity of grassroots institutions is unable to match their functional needs. (YZ-9)

Obviously, the severe shortage of health resources is a key factor constraining low-level medical institutions from fulfilling their functions in HDT. In practice, to balance this resource shortage, resource sharing between high-level and low-level medical institutions has been advocated by HDT for a long time. The sinking of health resources has long been desired. However, taking talents as an example, what often complicates this process is the presence of specialized doctors. GPs are talent resources that can match well with the function of primary health services. In contrast, specialists with certain expertise are of limited help in upgrading the comprehensive skills of primary health workers in prevention, health care, and rehabilitation. This has resulted in the current capacity of primary health provision still failing to match its function. A large number of participants believed that the current allocation of health resources is still a clear “inverted pyramid.” This configuration cannot match the actual function that primary medical institutions have been designed to have in HDT.

There is a serious imbalance in the allocation of health resources among the three levels of medical institutions. The shortage at the grassroots level is obvious and has affected the accessibility and equity of health care provision there. Mismatched resources inevitably lead to a mismatch in the flow of patients. (RM-2)

Doctors from high-level medical institutions are required to go to the grassroots and take up postings. In some cases, it’s even mandatory. However, what the grassroots really need are GPs who can upgrade the comprehensive skills of the grassroots in prevention, health care, and rehabilitation. Those who go down from high-level medical institutions are often specialists. Such a resource sinking still doesn’t match the requirement of HDT for the grassroots. (YZ-8)

#### 3.2.3 Deep-rooted health policy reforms not yet in place

Participants hold the view that establishing a “hierarchy of health service provision based on the occurrence pattern of diseases” is crucial for HDT. Different levels of medical institutions will cooperate through different levels of service provision. This will contribute to achieving “the intensification of limited health service resources and the maximization of functions.” This is regarded as the optimal solution to the dilemma of “difficult” and “expensive” access to health services given the current limited health resources. However, many participants also pointed out that this is a “premise based on ideal assumptions” since the construction of HDT, to a large extent, also depends on the coordinated adjustment of other related health policies. At present, the predicament of health resources allocation according to administrative levels, the talent sinking caused by the constraints of establishments and practice, and the unreasonable salary standards are all manifestations of the mismatch between the health policy environment and the construction of HDT. These not only reduce the service supply capacity of low-level medical institutions but also impede the efficient rationalization of the service supply of high-level institutions.

It's an undeniable fact that most highly educated and qualified doctors are gathered in high-level medical institutions. If there’s no supporting policy to truly encourage and allow them to move around, upgrading primary care services might just be a slogan. (YZ-2)

Many policies are mismatched and need adjustments, not just about resources and technology. A lot of similar policies have led to the current mismatch. Obviously, deep-rooted health policy reforms are still not in place. (DQ-1)

It has also been argued that the vertically divided management policy of medical institutions is incongruous with the current demand for HDT. Such a policy can result in cross-functionality and duplication of resources. Owing to factors like “long absence of an insurance system in the past,” “policies aimed at allowing medical institutions to make profits and generate income,” and “lack of government supervision,” the urban health care system has a significant concentration of high-quality resources. High-level medical institutions are inclined to deviate from their functional roles and operate in a wide range of profit-seeking areas. The absence of a mandatory gatekeeper system further undermines the patient-retention capacity of low-level medical institutions. The competitive situation between low-level and high-level medical institutions has created a major obstacle to the realization of “primary care and two-way referral.” As this cycle repeats, the dilemma of constructing HDT caused by the policy environment becomes more pronounced.

The mismatch in the hierarchical healthcare system started with the market-oriented reform in the healthcare field. It’s all because of the uncoordinated nature of a bunch of systems, like the healthcare insurance system, the health resource allocation system, and the system for training GPs. Competition has taken the place of cooperation among healthcare institutions. (YZ-5)

The government still hasn’t properly corrected market failures and compensated for the market’s shortcomings. Instead, it has followed market forces and directed a significant number of financial resources to high-level medical institutions. Without fundamental adjustments to the policy environment, it will be tough for medical institutions at all levels to fulfill their functions in the hierarchical healthcare system. (LS-2)

## 4 Discussion

The construction of HDT is a top priority in China’s health system reform (1). However, it has long been less effective than expected. This study reveals that this inefficiency is partly due to the mismatch between supply and demand. To address these issues, the present study suggests restoring residents’ confidence in low-level medical institutions’ service provision for realizing initial diagnosis at the grassroots level, establishing a positive health resource–allocation pyramid from lower to higher levels to enhance primary health care institutions’ service capacity, and adjusting the policy environment to sort out and reform deep-rooted policy contradictions and rationalize the functional positioning and matching relationship of medical institutions at all levels in HDT.

First, “initial diagnosis in primary medical institutions” lies at the core of HDT construction (15). Residents’ “chasing high-level medical [services]” behaviors directly reflect their lack of confidence in the provision of grassroots-level health services. This might be the key reason why “initial diagnosis in primary medical institutions” of HDT cannot be effectively implemented. Thus, it is of the utmost importance to rebuild residents’ related confidence (35). The inevitable connection between confidence and behavior has long been established. Particularly in the health field, behavioral confidence can directly influence patients’ health behaviors, health outcomes, and even the doctor–patient relationship (36). Some studies have indicated that residents’ confidence is mostly based on their experiences (37). Therefore, to rebuild residents’ confidence in the provision of grassroots-level health services, targeted efforts are needed to optimize service links and eliminate negative experience-related factors. Currently, the grassroots supply shows deficiencies in various aspects, including both variety and quality (38). Hence, every effort should be made to expand the variety and quantity of grassroots health service provision in HDT (39). Low-level medical institutions should be actively encouraged to make adaptive adjustments in light of changes in the local population’s disease spectrum. Only in this way can they effectively cover local medical service demands within their functional positioning in HDT. Furthermore, the demand for the quantity of health supply within the service scope should be scientifically estimated and then rationally coordinated and stockpiled to improve the efficiency and effectiveness of service provision. At the same time, every effort should be made to upgrade the quality of grassroots health services. With the increasing diversification of residents’ demand for health services provision, many factors, such as the experience, environment, and effect of medical services, can affect their confidence in grassroots institutions (39, 40). Therefore, efforts should be concentrated on building better health software and hardware facilities; standardizing service processes; and accelerating the establishment of influential quality service projects, such as the “contracted family doctor” program. This is crucial for rebuilding the “reputation” of grassroots health services among residents and enhancing their confidence (41, 42). For example, by emphasizing the professional guidance function of family doctors, current contracted family doctors can actively participate in the daily management and intervention of residents’ health and realize the functions of diagnosis, screening, and treatment of common and frequent diseases in practice (27). This is of great significance for ensuring the effective implementation of “initial diagnosis in primary medical institutions” and promoting the effective utilization of grassroots medical resources in HDT (43). At the same time, it can prevent a large number of high-quality medical resources in HDT that should be dealing with acute and critical illnesses from being crowded out (44).

Second, a “top-narrow and bottom-wide” health resource–allocation system is needed to address the mismatch in the functional positioning of HDT. As is known, rational allocation of health resources is the intention of HDT (45); such determines the service-provision ability and efficiency in HDT and the suitability of medical institutions’ functional positioning at all levels, and it is also key to solving the current shortage (46). According to HDT’s positioning of different levels of medical institutions, grassroots institutions should provide the most services, cover the most people, and be used the most. In contrast, high-level institutions should focus on mastering sophisticated technology and treating complex diseases. Generally, demand for high-level services is lower than that at the grassroots level (47). As such, existing resource allocation should be skewed toward low-level institutions—specifically, efforts should target accelerating the training of sufficient GPs with preventive treatment and management capabilities to meet grassroots needs, ensuring that the grassroots have a high density of general practice clinics for diagnosis, prevention, and treatment. After all, this is the first line of defense for safeguarding the health rights and interests of the population and has the greatest impact on people’s health (48). Hence, grassroots should be the focus of resource allocation. For high-level medical institutions in HDT, considering the incidence pattern of critical illnesses, resources should be targeted to help them solve key and/or important health issues (29). More efforts should be made to increase the number and quality of specialized doctors in high-level institutions (44). Specialized general hospitals should be set up in specific regions in a focused way to ensure that high-level health service institutions can cover patients with acute and critical illnesses in a large enough area and provide them with highly sophisticated diagnostic and treatment services. However, regulations should be strengthened to restrain the current blind expansion, alteration, and construction of new high-level medical institutions. Related argumentation and supervision should be strictly enhanced. These are important for completing an “upper-narrow and lower-wide” health resource–allocation system to meet the policy needs of HDT.

Finally, making efforts to adjust the existing policy environment and resolve some deeply ingrained policy contradictions serves as one guarantee for addressing the mismatch (49). Institutional barriers within the policy environment are regarded as the root cause of the structural contradiction in healthcare resource allocation and have a profound impact on residents’ confidence when they are seeking medical care (50). Current research has revealed various impacts of existing policies on HDT from different aspects, such as healthcare personnel, finances, materials, technology, and information (51). Therefore, based on the results of this study, the policy environment should actively explore a series of reform measures in the future. It should modify the establishment, treatment, and input rules according to different institutional levels to facilitate the effective mobility of high-level healthcare talents in HDT (42). An effective coordination mechanism for practitioners’ qualifications, insurance reimbursement, and fee payment standards should be explored to ensure the smooth flow of triage and referral channels. Channels and ways for information sharing, resource exchange, technology transfer, and training and teaching should also be explored to effectively enhance the service supply capacity of grassroots healthcare institutions. Additionally, a similar “gatekeeper” system should be explored to guide the development of grassroots health service programs such as “contracted family doctors” and to provide orderly guidance for residents’ unregulated consultation behaviors (52). Furthermore, deepening the reform of the personnel allocation system to guide high-quality healthcare resources to flow to rural and grassroots areas (31), reforming the personnel allocation system to facilitate the sinking of high-quality resources, clarifying the developmental positioning of all-level medical institutions, and improving HDT standards and procedures are also warranted (53). Only by rationally addressing policy contradictions in the policy environment can we provide a practical guarantee for HDT development.

## 5 Limitations

Although this study has some strengths, it also has several limitations. First, the snowball sampling list was derived from the participants. Sample size and sample heterogeneity may be limited. As a result, the representativeness of the conclusions might be affected. Second, limited by the inherent inadequacies of qualitative research methods, possible bias could have led to inappropriate conclusions if the participants tried to emphasize some highly subjective opinions or if they focused on their own interests. Third, also limited by the inherent inadequacies of qualitative research methods, the observed associations between residents’ trust and HDT outcomes are correlational rather than causal. Longitudinal or interventional studies are therefore needed to disentangle the direction of these relationships and to test causal pathways. Fourth, given its purposive study design, this investigation was unable to systematically measure many potential confounders, such as socioeconomic gradients, culturally mediated health beliefs, and geographic access barriers, thereby influencing our findings. Future mixed-methods studies that incorporate these variables are warranted. Finally, all interviews were conducted in ZX City, a county-level municipality in central Hubei Province. Consequently, the transferability of the grounded theory to cities with different health resource structures remains to be tested. Thus, additional studies with expanded sample sizes and/or that are conducted using some objective methodologies and/or that consider the perspective of the public are needed. However, these limitations do not abrogate the insights generated by the present study with regard to clarifying the mismatch between HDT construction and related reasons.

## 6 Conclusion

This study found that there are notable mismatches between residents’ “pursuit of high-level medical care” and HDT’s requirement of “initial diagnosis at [the] grassroots level,” between service supply capacity and the functional positioning of medical institutions in HDT, and between the policy environment and HDT’s construction needs. The main reasons for these mismatches include residents’ lack of confidence, an improper allocation of health resources, and the absence of deep-rooted health policy reforms. Thus, this study suggests restoring residents’ confidence in low-level medical institutions’ service provision for realizing initial diagnosis at the grassroots level, establishing a positive health resource allocation pyramid from lower to higher levels to enhance primary health care institutions’ service capacity, and adjusting the policy environment to sort out and reform deep-rooted policy contradictions and rationalize the functional positioning and matching relationship of medical institutions at all levels in HDT.

## Data Availability

The raw data supporting the conclusions of this article will be made available by the authors, without undue reservation.
